# Nursing in America

**Published:** 1888-03-17

**Authors:** 


### NURSING IN AMERICA.

"Gambier," with seven years' nursing experience, including midwifery, is anxious to obtain an appointment in Canada or the United States. Could she do so before leaving England, and to whom should she apply for information?

